# Mdivi-1 induces spindle abnormalities and augments taxol cytotoxicity in MDA-MB-231 cells

**DOI:** 10.1038/s41420-021-00495-z

**Published:** 2021-05-20

**Authors:** Chieh-Ting Fang, Hsiao-Hui Kuo, Chia-Jung Yuan, Jhong-Syuan Yao, Ling-Huei Yih

**Affiliations:** 1 Institute of Cellular and Organismic Biology, Academia Sinica, Taipei, Taiwan; 2 Institute of Biomedical Science, National Tsing Hua University, Hsinchu, Taiwan

**Keywords:** Cell death, Chemotherapy

## Abstract

Taxol is a first-line chemotherapeutic for numerous cancers, including the highly refractory triple-negative breast cancer (TNBC). However, it is often associated with toxic side effects and chemoresistance in breast cancer patients, which greatly limits the clinical utility of the drug. Hence, compounds that act in concert with taxol to promote cytotoxicity may be useful to improve the efficacy of taxol-based chemotherapy. In this study, we demonstrated that mdivi-1, a putative inhibitor of mitochondrial fission protein Drp1, enhances the anticancer effects of taxol and overcomes taxol resistance in a TNBC cell line (MDA-MB-231). Not only did mdivi-1 induce mitotic spindle abnormalities and mitotic arrest when used alone, but it also enhanced taxol-induced antimitotic effects when applied in combination. In addition, mdivi-1 induced pronounced spindle abnormalities and cytotoxicity in a taxol-resistant cell line, indicating that it can overcome taxol resistance. Notably, the antimitotic effects of mdivi-1 were not accompanied by prominent morphological or functional alterations in mitochondria and were Drp1-independent. Instead, mdivi-1 exhibited affinity to tubulin at μM level, inhibited tubulin polymerization, and immediately disrupted spindle assembly when cells entered mitosis. Together, our results show that mdivi-1 associates with tubulin and impedes tubulin polymerization, actions which may underlie its antimitotic activity and its ability to enhance taxol cytotoxicity and overcome taxol resistance in MDA-MB-231 cells. Furthermore, our data imply a possibility that mdivi-1 could be useful to improve the therapeutic efficacy of taxol in breast cancer.

## Introduction

Triple-negative breast cancer (TNBC) cells lack expression of the estrogen receptor, progesterone receptor, and HER2 receptor, and the disease accounts for ~10–20% of all diagnosed breast cancers. Due to the lack of receptors as drug targets, TNBC is the most refractory subtype of breast cancer, and it is associated with worse overall survival, higher risks of recurrence and metastasis, and poorer prognosis than other subtypes^1–3^. Currently, cytotoxic chemotherapy remains the primary treatment for TNBC.

Standard chemotherapeutic regimens for breast cancers include the use of taxane compounds as monotherapies or in combination with other therapeutics^1,2^. The prototypical taxane, taxol, is known to target tubulin, stabilize microtubule polymerization and disrupt mitotic spindle assembly to exert its antimitotic and anti-proliferative effects; taxol has remained a first-line chemotherapeutic for numerous cancers since its identification decades ago^4^. However, taxol treatment is often associated with severe side effects, such as hypersensitivity reactions, peripheral neuropathy and cardiotoxicity, which limit the dosage and sometimes necessitate a halt to treatment^5–8^. In addition, taxol resistance can be developed through altered microtubule dynamics^9,10^, differential tubulin isoform expression^11,12^, or mutations in tubulin or associated proteins that perturb taxol–microtubule interaction^13,14^, or through alterations in signaling pathways involved in cell proliferation^15,16^, apoptosis^17,18^, DNA repair^19^, metabolic reprogramming^20,21^, immunomodulation^22^, and xenobiotic export^23,24^. These resistance mechanisms not only lead to treatment failures but may also further drive cancer progression and disease recurrence, contributing to overall poor prognosis for patients^1,25,26^. Thus, treatment-limiting side effects and chemoresistance present significant obstacles in the clinical application of taxanes^2,27^.

To ameliorate toxic side effects and overcome resistance to taxol-based chemotherapy in breast cancers, new treatments combining taxol with other therapeutics are currently under extensive investigation. Such combinations may reduce the dosage and/or enhance the anticancer effects of taxol. For example, inhibitors of tyrosine kinases, PI3K/AKT/mTOR constituents, PARP1/2, and immune checkpoint proteins have been examined in clinical trials to delineate their effects in combination with taxol^1–3^. Recently, the combination of albumin-bound taxol and atezolizumab, which targets PD-L1, became one of the first FDA-approved first-line combination therapies^28^. While the outcomes of clinical trials and elucidation of chemoresistance mechanisms are still awaited for most combination therapies, the search for cytotoxic compounds to improve the efficacy of taxol-based chemotherapy remains a priority.

Mdivi-1 is a putative inhibitor of mitochondrial fission that was reported to inhibit the GTPase activity of Drp1 in yeast and induce mitochondrial hyperfusion and elongation in mammalian cells^29–31^. More recently, mdivi-1 has been reported to induce apoptosis by itself or to enhance chemotherapy-induced apoptosis in several cancer cells via Drp1-dependent and Drp1-independent mechanisms^32–36^. Thus, mdivi-1 may display antitumor effects that involve distinct mechanisms, suggesting that it may have potential use as a component of combination chemotherapies. To test this idea, we examined whether and how mdivi-1 acts to improve the efficacy of taxol in a TNBC cell line, MDA-MB-231. We found that mdivi-1 enhances taxol cytotoxicity and overcomes taxol resistance in MDA-MB-231 cells. In addition, mdivi-1 displays antimitotic effects that appear to be independent of Drp1 and mitochondria. Rather, mdivi-1 may associate with tubulin, inhibit tubulin polymerization and disrupt mitotic spindle integrity to enhance taxol cytotoxicity and to overcome taxol resistance.

## Results

### Mdivi-1 enhances the cytotoxicity of taxol and overcomes taxol resistance in MDA-MB-231 cells

**Fig. 1:**
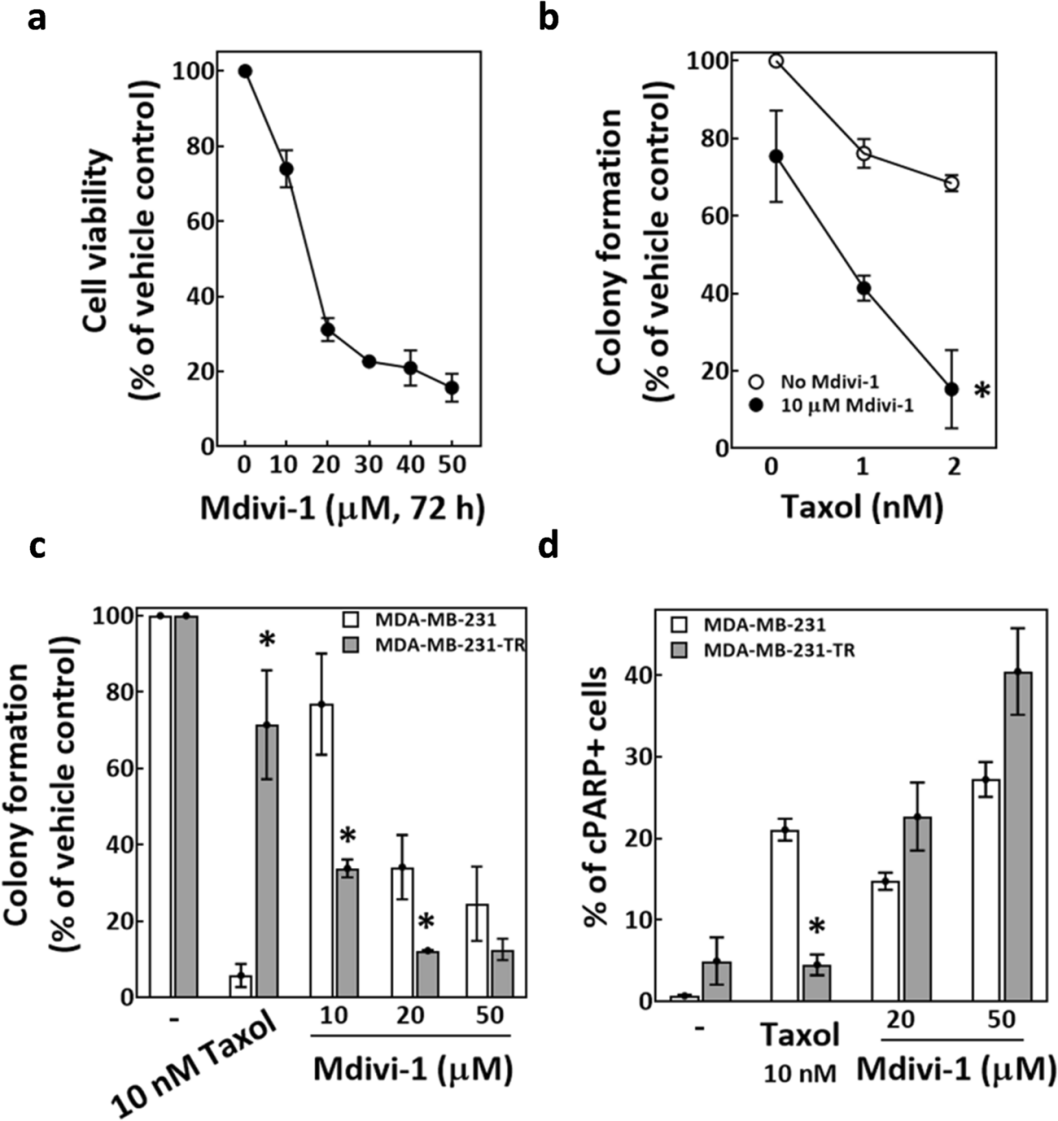
Mdivi-1 enhances cytotoxicity of taxol and overcomes taxol resistance in MDA-MB-231. **a** Cytotoxicity of mdivi-1 in MDA-MB-231 cells. Cells were treated for 72 h with mdivi-1 at the indicated concentrations and then subjected to trypan blue exclusion assay. The percentages of cell viability relative to the vehicle control are shown as mean ± SD from at least three independent experiments. **b** Mdivi-1 enhanced the cytotoxicity of taxol. Cells were treated either with taxol alone or in combination with mdivi-1 at the indicated concentration for 24 h and then subjected to colony formation assay. The percentages of colonies formed compared to the vehicle control are shown as mean ± SD from four independent experiments. *p < 0.05 comparing 10 μM mdivi-1-treated groups to no mdivi-1-treated groups by two-way ANOVA. **c** and **d** The taxol-resistant MDA-MB-231-TR cells remained sensitive to mdivi-1. MDA-MB-231 and the resistant MDA-MB-231-TR cells generated as described were treated as indicated for 24 h and then used in the colony formation assay (**c**) or cells were treated for 72 h and subjected to flow cytometry analysis for cleaved-PARP-positive (cPARP+) cells (**d**). The percentages of colonies formed relative to vehicle control and percentages cPARP+ cells are shown as mean ± SD from three independent experiments. *p < 0.05 comparing MDA-MB-231-TR to MDA-MB-231 by Student’s *t* test.

To begin exploring the potential of mdivi-1 to improve taxol-based chemotherapy, we first tested the viability of MDA-MB-231 TNBC cells treated with mdivi-1 at serial dosages; our aim was to determine an appropriate mdivi-1 concentration for combined treatment. Figure 1a shows the viability of MDA-MB-231 cells after mdivi-1 treatment for 72 h. Notably, 10 μM mdivi-1 only caused minor cytotoxicity in the culture. We then treated MDA-MB-231 cells with taxol plus 10 μM mdivi-1 and found that the combined treatment significantly reduced the colony formation ability of MDA-MB-231 cells compared to taxol treatment alone (Fig. 1b). In addition, according to the combination index (CI) theory of Chou–Talalay^37^, the CI of our combined treatments are smaller than 1, suggesting that mdivi-1 synergistically enhances the cytotoxicity of taxol in MDA-MB-231 cells. Since acquired chemoresistance and associated cancer relapse are documented undesirable outcomes following taxol treatment^38,39^, we established a taxol-resistant MDA-MB-231 cell line (MDA-MB-231-TR) and tested its response to mdivi-1. While MDA-MB-231-TR retained a high level of colony formation ability after taxol treatment alone, they were significantly more sensitive to mdivi-1 treatment alone than parental MDA-MB-231 cells (Fig. 1c). Furthermore, the level of cleaved-PARP (cPARP) in MDA-MB-231-TR cells was significantly lower after the 72 h treatment of taxol alone but was higher following 72 h mdivi-1 treatment alone compared to the respective levels in the parental line (Fig. 1d). These findings suggested that mdivi-1 induces apoptotic cell death more effectively in MDA-MB-231-TR than in MDA-MB-231. Together, these data indicated that mdivi-1 enhanced the cytotoxicity of taxol to overcome the resistance to taxol in MDA-MB-231 cells. Thus, mdivi-1 may have the potential to improve taxol-based chemotherapies.

### Mdivi-1 disrupts mitotic spindle assembly, enhances taxol-induced spindle abnormalities, and induces spindle defects in taxol-resistant cells

**Fig. 2:**
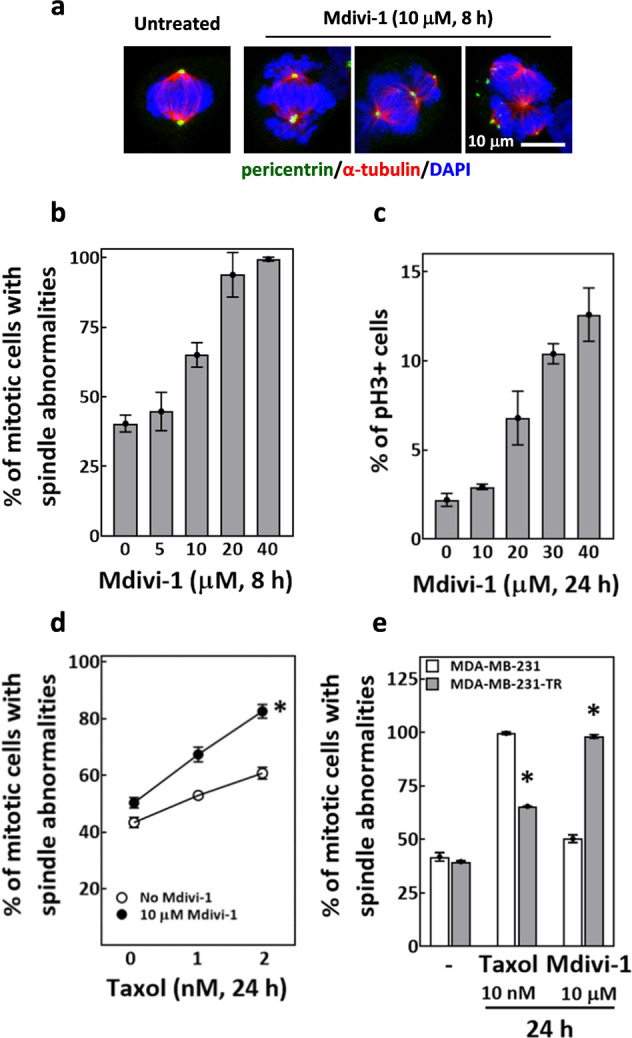
Mdivi-1 disrupts mitotic spindle assembly, enhances taxol-induced spindle abnormalities, and induces spindle defects in taxol-resistant cells. **a** Representative image of untreated cells with a normal mitotic spindle and mdivi-1-treated cells with abnormal mitotic spindles. Cells were fixed and immunostained for pericentrin (green), α-tubulin (red), and chromosomes (blue). **b** Mdivi-1 dose-dependently induced spindle abnormalities. MDA-MB-231 cells were treated for 8 h with the indicated concentrations of mdivi-1, then subjected to analysis of mitotic spindles. Percentages of mitotic cells with spindle abnormalities are shown as mean ± SD from at least three independent experiments. **c** Mdivi-1 induced mitotic arrest. Cells were treated with mdivi-1 as indicated and subjected to flow cytometry analysis for phospho-histone H3-positive (pH3+) cells. The mean ± SD from three independent experiments is shown. **d** Mdivi-1 further enhanced the spindle abnormalities induced by taxol. Cells were treated either with taxol alone or in combination with mdivi-1 at the indicated concentration for 24 h and then subjected to analysis of mitotic spindles. The mean ± SD from at least three independent experiments is shown. *p < 0.05 comparing 10 μM mdivi-1-treated groups to no mdivi-1-treated groups by two-way ANOVA. **e** The taxol-resistant MDA-MB-231-TR cells remained sensitive to mdivi-1-induced spindle abnormalities. MDA-MB-231 and the MDA-MB-231-TR cells were treated as indicated for 24 h and then subjected to analysis of mitotic spindles. Percentages of mitotic cells with spindle abnormalities are shown as mean ± SD from three independent experiments. *p < 0.05 comparing MDA-MB-231-TR to MDA-MB-231 by Student’s *t* test.

We next examined whether mdivi-1 can augment the antimitotic effects of taxol. We found that 8-h treatments of mdivi-1 alone in MDA-MB-231 cells could dose-dependently increase the percentage of mitotic cells with spindle abnormalities (Fig. 2a and b). With regard to the cell cycle, mdivi-1 treatment for 24 h induced a dose-dependent accumulation of phospho-histone H3 (p-H3)-positive cells (Fig. 2c). These observations suggested that mdivi-1 exerts antimitotic effects, as it disrupted mitotic spindle assembly and induced mitotic arrest in MDA-MB-231 cells. Furthermore, combined treatment of taxol and 10 μM mdivi-1 caused a further increase in mitotic cells with spindle abnormalities compared to taxol treatment alone (Fig. 2d), suggesting that mdivi-1 may enhance taxol-induced spindle abnormalities. In addition, the taxol-resistant MDA-MB-231-TR cell line showed only a minor effect of taxol-induced spindle abnormalities but a greater effect of mdivi-1-induced spindle abnormalities compared to parental MDA-MB-231 cells (Fig. 2e). Hence, MDA-MB-231-TR cells appear to be refractory to taxol-induced spindle abnormalities but more sensitive to mdivi-1-induced spindle abnormalities than MDA-MB-231 cells. Overall, these experiments showed that mdivi-1 disrupted mitotic spindle assembly and induced mitotic arrest; these antimitotic effects may further enhance taxol-induced spindle abnormalities and cytotoxic effects in MDA-MB-231 cells. In addition, the ability of mdivi-1 to induce cytotoxicity in MDA-MB-231-TR cells suggested that mdivi-1 and taxol cause cytotoxicity via different mechanisms.

### Mdivi-1 induces mitotic abnormalities independently of Drp1

**Fig. 3:**
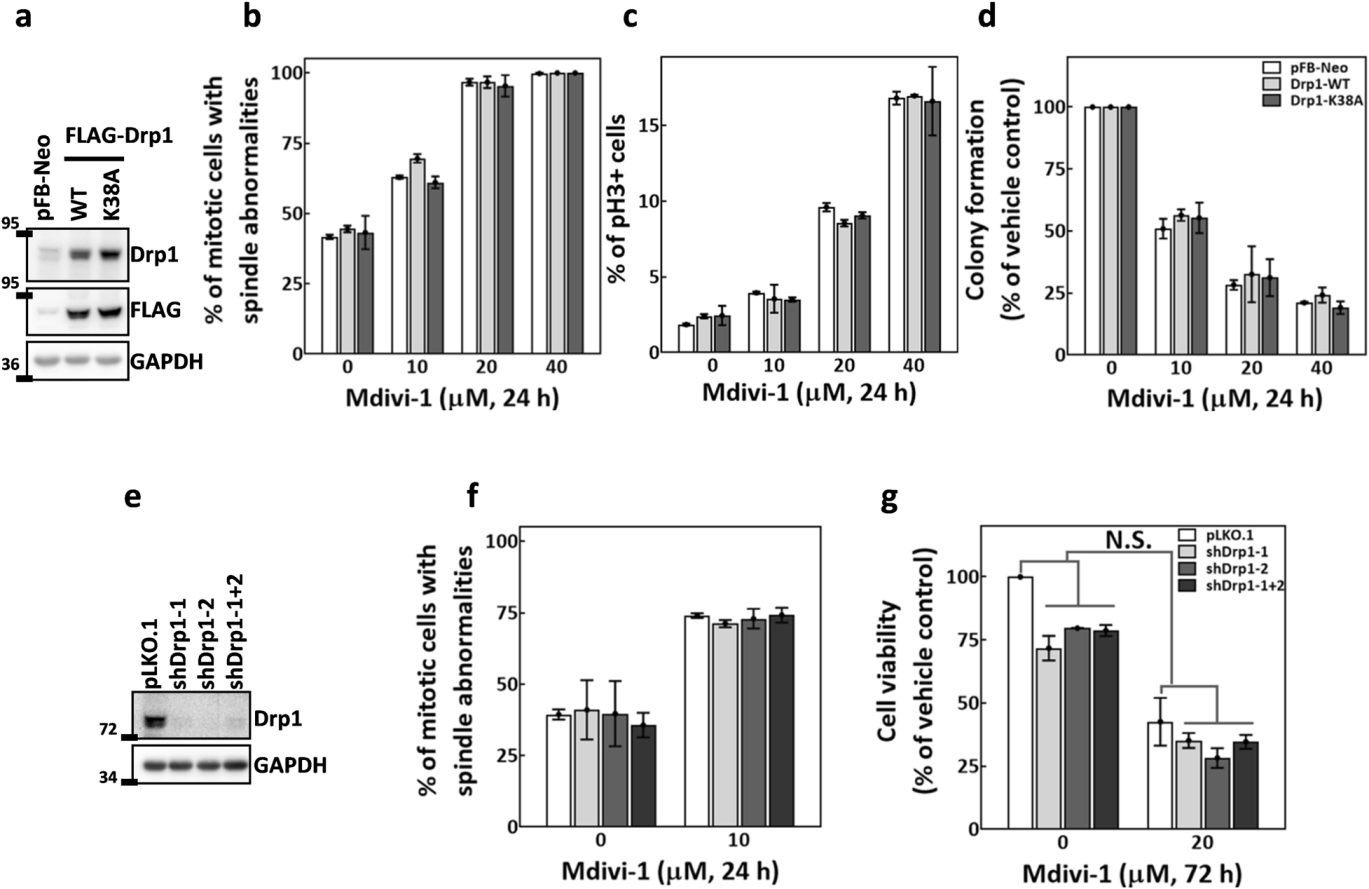
Mdivi-1 induces mitotic abnormalities independent of Drp1. **a** Western blot shows the overexpression efficiency of FLAG-Drp1-WT and -K38A. **b**–**d** Overexpression of Drp1 did not affect the antimitotic effects of mdivi-1. MDA-MB-231 cells harboring empty vector (pFB-Neo) or overexpressing wild-type (Drp1-WT) or dominant-negative mutant (Drp1-K38A) Drp1 were treated with indicated concentrations of mdivi-1 for 24 h and then subjected to analysis of spindle abnormalities (**b**), mitotic arrest (**c**), and colony formation ability (**d**). Data are presented as mean ± SD from three independent experiments. **e** Western blot shows the depletion efficiency of shRNA targeting Drp1. **f** and **g** Depletion of Drp1 did not affect the antimitotic effects of mdivi-1. MDA-MB-231 cells depleted of Drp1 were treated with the indicated concentrations of mdivi-1 for 24 h and then subjected to analysis of spindle abnormalities (**f**) or cells were treated for 72 h and then subjected to trypan blue exclusion assay for viability analysis (**g**). Data presented are mean ± SD from three independent experiments. N.S. no significance comparing 20 μM mdivi-1 to no mdivi-1 by two-way ANOVA.

Mdivi-1 was first reported in yeast to inhibit the GTPase activity of Drp1, a mitochondrial fission mediator^29^. We, therefore, investigated the possible involvement of Drp1 in mdivi-1-induced cytotoxicity by examining the antimitotic effects of mdivi-1 in cells with modulated Drp1 levels. As shown in Fig. 3a, the FLAG-tagged WT Drp1 (FLAG-Drp1-WT) and the FLAG-tagged dominant-negative mutant Drp1 (FLAG-Drp1-K38A)^40^ were overexpressed in MDA-MB-231 cells. Cells harboring empty vector pFB-Neo were used as a control. We found that the percentage of cells with spindle abnormalities (Fig. 3b), the p-H3-positive cells (Fig. 3c), and the colony formation ability (Fig. 3d) were all affected by escalating doses of mdivi-1 at similar levels in control, FLAG-Drp1-WT-overexpressing, and FLAG-Drp1-K38A-overexpressing cells. These data indicated that overexpression of WT or mutant Drp1 had no observable effect on mitosis progression in untreated cells or the antimitotic effects of mdivi-1.

We then proceeded to investigate whether Drp1 depletion in MDA-MB-231 cells alters the antimitotic effects of mdivi-1. Virions containing either of two Drp1-specific shRNAs (targeting different regions of *DNM1L*) or those containing both shRNAs were transduced into cells to deplete cellular Drp1; cells transduced with virions harboring empty vector pLKO.1 were used as a control. Cellular Drp1 was efficiently depleted by shRNA-mediated knockdown (Fig. 3e). We found that none of the depletion strategies altered the percentages of cells displaying spindle abnormalities in either untreated or mdivi-1-treated cells (Fig. 3f). In addition, while all of the Drp1 depletion strategies moderately reduced the viability of the untreated cells to 70% comparing to the control, none significantly altered the mdivi-1-induced reduction of viability (Fig. 3g). These observations suggested that Drp1 probably does not play an essential role in mitosis progression, cell proliferation, or mdivi-1-induced mitotic abnormalities in MDA-MB-231 cells.

### Mitochondrial morphology and functions are not significantly altered in cells with mdivi-1-induced mitotic defects

**Fig. 4:**
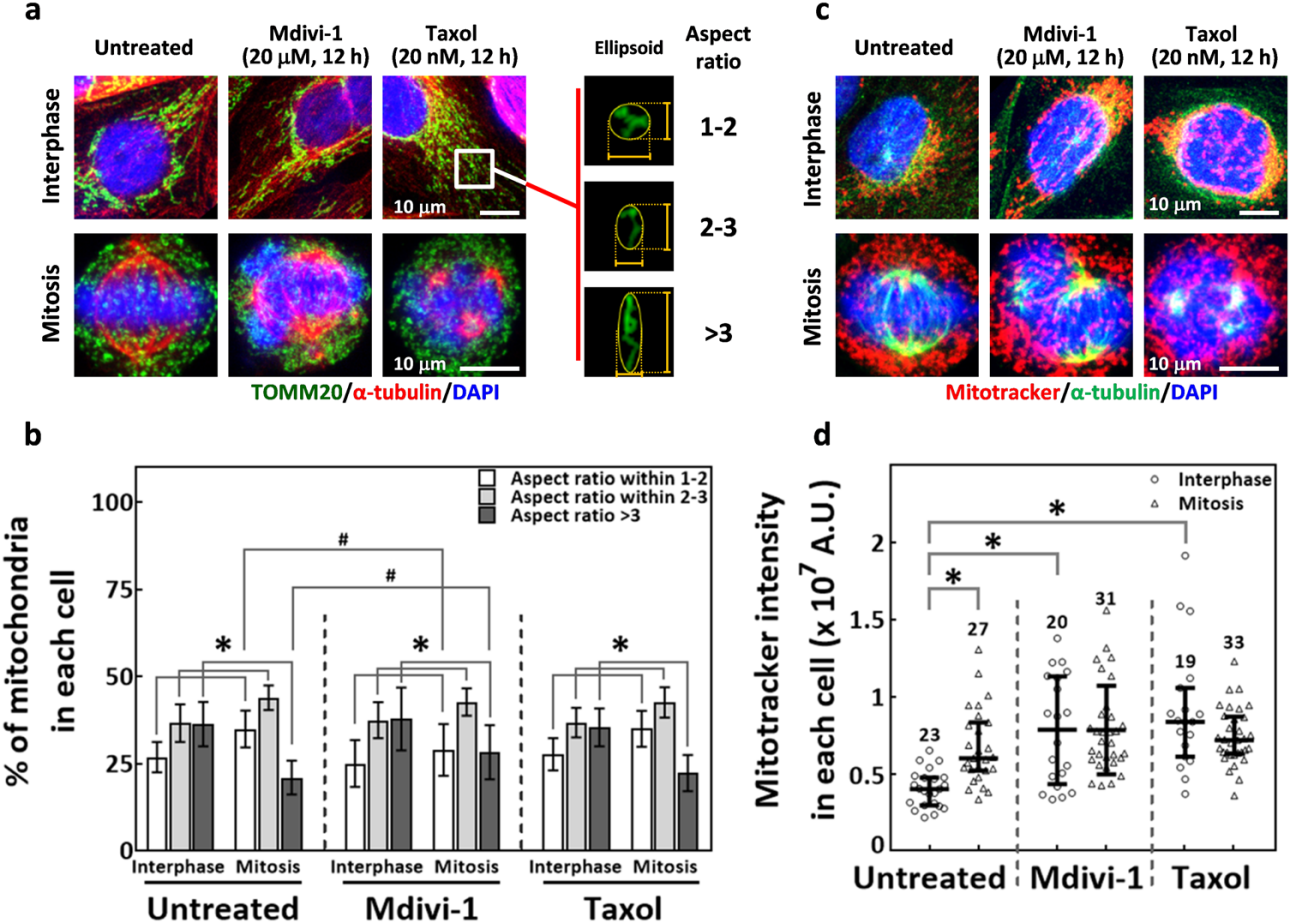
Mitochondrial morphology and functions are not significantly altered in cells with mdivi-1-induced mitotic defects. **a** (Left) Representative images show mitochondrial morphology in interphase (upper) and mitotic (lower) cells that were treated as indicated. Mitochondria were revealed by TOMM20 immunostaining (green), and the cells were counterstained for α-tubulin (red) and chromosomes (blue). (Right) The illustration depicts the level of mitochondrial fragmentation, as represented by the aspect ratio of circumscribed ellipsoids that were obtained as described. The ovals illustrate the circumscribed ellipsoids and the yellow lines delineate the longest and the shortest axis of the ellipsoid. The representative images show mitochondria with aspect ratios of 1-2, 2-3, and >3. **b** Mdivi-1 did not prominently alter the characteristic changes in mitochondria morphology during the G2-M transition. The percentages of mitochondria within each cell that exhibit the indicated aspect ratio are shown as mean ± SD from at least 20 cells collected from two independent experiments. *p < 0.05 comparing mitosis to interphase by Student’s *t* test. ^#^*p* < 0.05 comparing drug-treated groups to untreated by Student’s *t* test. **c** Representative images of interphase (upper) and mitotic (lower) cells that were treated as indicated. Cells were stained with MitoTracker Deep Red (mitotracker, red) and counterstained for α-tubulin (green) and chromosomes (blue). **d** Mdivi-1 did not prominently affect the mitotracker intensity in arrested abnormal mitotic cells. The relative total mitotracker intensity in each cell was measured as described. The scatter plot shows the interquartile distribution of the mitotracker intensity in cells collected from two independent experiments; numbers indicate the cells measured. *p < 0.05 by Mann–Whitney Rank Sum test.

Mdivi-1 has been widely reported to enhance mitochondrial fusion, which induces mitochondrial elongation^29,31^. Since our results showed that mdivi-1-induced mitotic abnormalities might be independent of Drp1, we examined whether mdivi-1 effects on spindle assembly might involve alterations in mitochondrial morphology. We performed immunofluorescence staining of TOMM20, a mitochondrial marker protein^41^, to examine mitochondrial morphology in MDA-MB-231 cells treated with 20 μM mdivi-1; this concentration induced considerable spindle abnormalities in our previous experiments (Fig. 2b). Mitochondria in cells treated with taxol were examined as a control. We first observed that mitochondria in untreated mitotic cells were more fragmented and less elongated than those in interphase cells (Fig. 4a, Untreated), a phenomenon that was reported previously^42,43^. To infer the level of mitochondrial fragmentation, we measured the aspect ratios of ellipsoids that circumscribed the mitochondria and classified these aspect ratios into three size ranges: 1–2, 2–3, and >3 (Fig. 4a right panel). As shown in Fig. 4b, Untreated, the percentages of mitochondria with aspect ratio in ranges 1–2 and 2–3 were increased and those with aspect ratio >3 were decreased in mitotic cells compared to interphase cells. These data supported the general conclusion that mitochondria become fragmented and less elongated during mitosis. In cells treated for 12 h with mdivi-1, we saw that mitochondria displayed similar changes; the mdivi-1-treated mitotic cells showed higher percentages of mitochondria with aspect ratios in ranges 1–2 and 2–3 and lower percentages of those >3 compared to mdivi-1-treated interphase cells (Fig. 4a, Mdivi-1 and Fig. 4b, Mdivi-1). We then compared the mdivi-1-treated cells to the untreated cells and found that the fractions of mitochondria within the three aspect ratio ranges were distributed similarly between untreated and mdivi-1-treated interphase cells, suggesting that 20 μM mdivi-1 does not likely induce mitochondria fusion in interphase MDA-MB-231 cells. Furthermore, a slightly lower percentage of mitochondria with aspect ratio in 1–2 and a slightly higher percentage of those >3 were observed in mdivi-1-treated mitotic cells compared to untreated mitotic cells (Fig. 4b). These observations suggested that despite significant spindle abnormalities, the mitochondria under mdivi-1 treatment still displayed normal morphological changes from interphase to mitosis, although the level of fragmentation during mitosis was slightly decreased. In addition, 12-h treatment with taxol-induced spindle abnormalities without changing mitochondrial morphology in G2-M transition, as the distribution of mitochondria within three aspect ratio ranges was similar to untreated cells during both interphase and mitosis. (Fig. 4a, Taxol and Fig. 4b, Taxol). Thus, taxol-induced spindle abnormalities were not accompanied by alterations in mitochondrial morphological changes. Together, these findings indicated that mitochondrial morphology is not significantly altered in mitotic cells with mdivi-1- or taxol-induced spindle abnormalities.

To study whether mdivi-1-induced mitotic defects were correlated with functional alterations in mitochondria, we analyzed mitochondrial membrane potential by staining cells with MitoTracker Deep Red, a mitochondrial membrane potential-indicating dye^44^. We observed in untreated cells that the intensity of MitoTracker Deep Red was strongly enhanced in mitotic cells compared to interphase cells (Fig. 4c, Untreated and Fig. 4d), suggesting a physiological elevation of the mitochondrial membrane potential during mitosis. Mdivi-1 and taxol treatment both enhanced the intensity in the interphase cells compared to the untreated cells (Fig. 4c, Interphase, Mdivi-1 and Taxol, and Fig. 4d), suggesting that mdivi-1 and taxol induced an elevation in the mitochondrial membrane potential during interphase. However, the mitotracker intensities in mdivi-1-arrested and taxol-arrested mitotic cells were similar to that in untreated mitotic cells (Fig. 4c, Mitosis, Mdivi-1, and Taxol, and Fig. 4d), suggesting that the mitochondrial membrane potential was not altered in mitotic cells with mdivi-1-induced or taxol-induced spindle defects. The lack of significant alterations in mitochondrial morphology and membrane potential by mdivi-1-induced or taxol-induced arrest of mitotic cells led us to conclude that mdivi-1-induced and taxol-induced mitotic defects are not likely to be associated with changes in mitochondrial morphology or function.

### Mdivi-1 associates with tubulin and inhibits tubulin polymerization

**Fig. 5:**
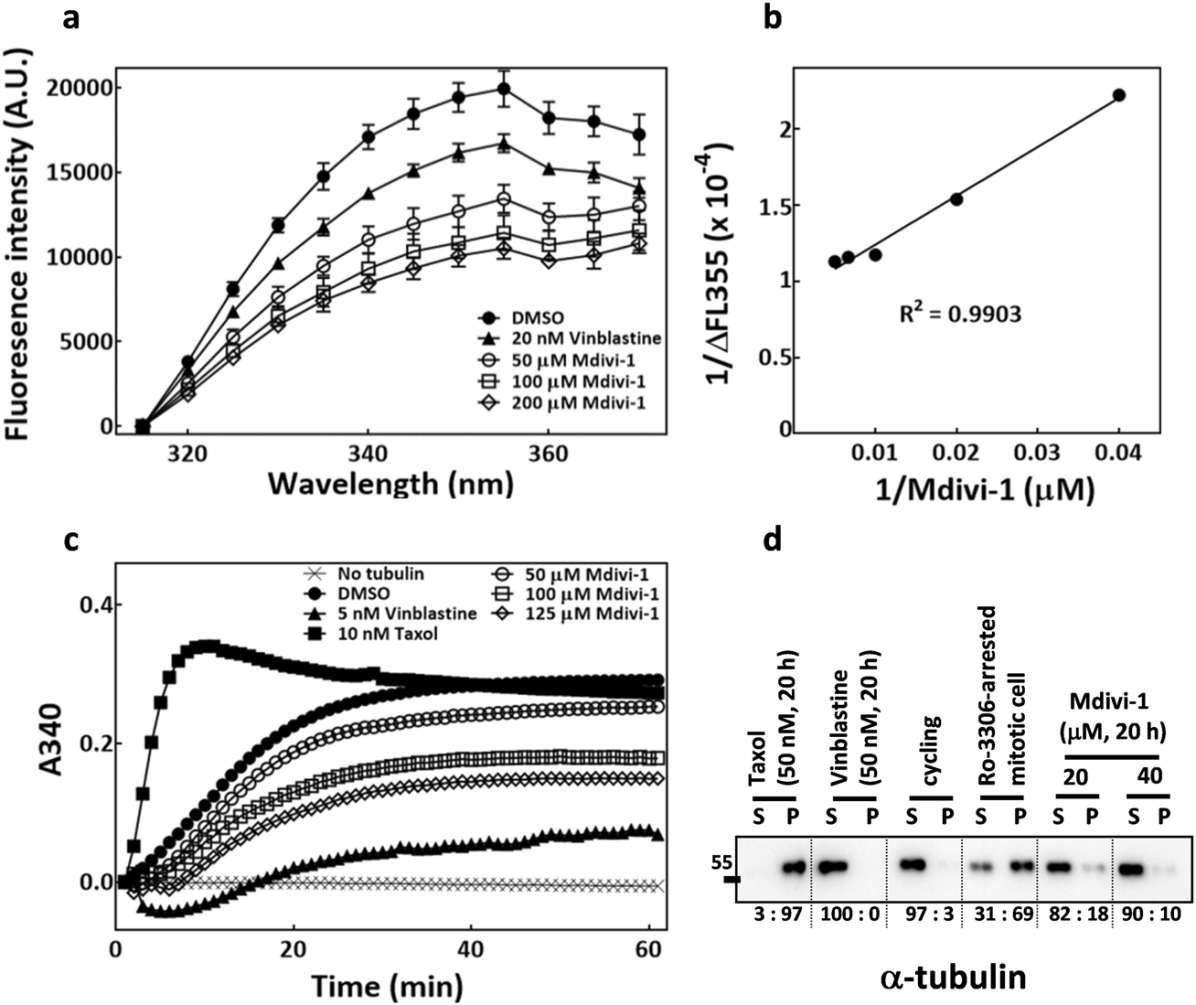
Mdivi-1 associates with tubulin and inhibits tubulin polymerization. **a** Mdivi-1 quenched the intrinsic tryptophan fluorescence of tubulin. The effect of mdivi-1 at indicated concentrations on intrinsic tryptophan fluorescence of tubulin was measured as described. The excitation wavelength was 295 nm. The effect of tubulin polymerization inhibitor vinblastine was also measured as a positive control. Means ± SD from three independent experiments is shown. **b** The mean reductions of tryptophan fluorescence at 355 nm (ΔFL355) induced by various concentrations of mdivi-1 from (**a**) are expressed as a double reciprocal plot, yielding a regression line with *R*^2^ value of 0.99. **c** Mdivi-1 inhibited tubulin polymerization in vitro. In vitro tubulin polymerization was carried out in the absence or presence of various concentrations of mdivi-1, and the level of polymerized tubulin was estimated by the absorbance at 340 nm (A340). The effects of taxol and vinblastine were also measured as positive and negative controls, respectively. The means of the three experiments are shown. **d** Mdivi-1 inhibited tubulin polymerization in arrested abnormal mitotic cells. A cellular tubulin polymerization assay was performed as described, and western blot was used to analyze the level of monomer tubulin in the supernatant (S) and polymerized tubulin in the pellet (P) of the lysate. The numbers underneath the blot indicate the mean percentages (two experiments) of each tubulin fraction relative to the total tubulin content (monomer + polymerized tubulin).

Based on our observations that mdivi-1 seems to induce spindle abnormalities and cytotoxicity independent of Drp1 and mitochondrial dynamics, we speculated that mdivi-1 may disrupt the mitotic spindle by altering microtubule dynamics. We first investigated whether mdivi-1 binds to tubulin by measuring its ability to quench the intrinsic tryptophan fluorescence of tubulin. Similar to the tubulin depolymerizing drug vinblastine, we found that mdivi-1 at μM concentrations reduced the intrinsic tryptophan fluorescence in a dose-dependent manner, with the maximum detected reduction at 355 nm (Fig. 5a). The change in the fluorescence emission at 355 nm (∆FL355) was calculated for each mdivi-1 concentration and then plotted on a double reciprocal plot (Fig. 5b), yielding a regression line with an *R*^2^ value of 0.99 and suggesting that mdivi-1 may associate with tubulin in a dose-dependent manner. Importantly, the mdivi-1 concentrations required to quench the intrinsic tryptophan fluorescence of tubulin correspond to those that induced significant spindle abnormalities in MDA-MB-231 cells. We then examined whether mdivi-1 could alter tubulin polymerization in vitro. As shown in Fig. 5c, both the rate and extent of tubulin polymerization were strongly enhanced by the tubulin stabilizing drug taxol, but both were dose-dependently decreased by mdivi-1 or the tubulin depolymerizing drug, vinblastine. Though midivi-1 did not show significant effects at concentrations below 50 µM in the in vitro assays (data not shown), these data suggested that mdivi-1 may directly bind to tubulin and inhibit tubulin polymerization.

To validate the inhibitory effects of mdivi-1 on tubulin polymerization in MDA-MB-231 cells, we performed a cellular tubulin polymerization assay, as previously described^45^. As shown in Fig. 5d, the tubulin extracts from cycling cells were primarily comprised of monomer, whereas nearly 70% of tubulin from the mitotic cells collected after Ro-3306 block and release was in the polymerized form. Accordingly, tubulin predominantly existed in a polymerized form in taxol-arrested mitotic cells and in a monomer form in vinblastine-arrested mitotic cells. Importantly, compared to the Ro-3306-blocked-and-released mitotic cell, the fractions of polymerized tubulin were significantly and dose-dependently reduced in the mitotic cells arrested by 20 μM mdivi-1 (18%) and 40 μM mdivi-1 (10%). Together, these data indicated that mdivi-1 can associate with tubulin, and it inhibits tubulin polymerization both in vitro and in MDA-MB-231 cells.

### Mdivi-1 sensitizes microtubules to cold-induced disassembly and immediately disrupts spindle assembly upon mitosis entry

**Fig. 6:**
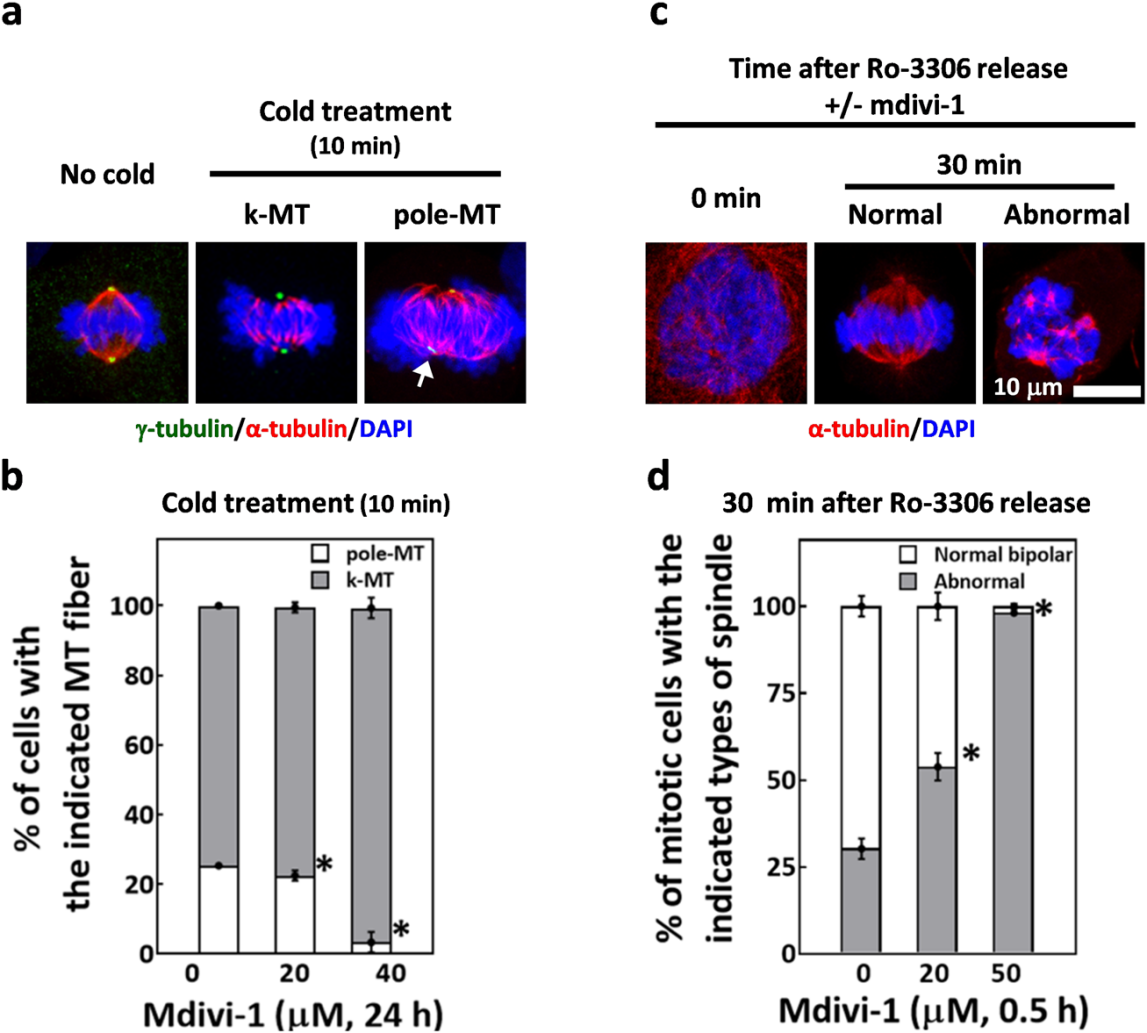
Mdivi-1 sensitizes microtubules to cold-induced disassembly and immediately disrupts spindle assembly upon mitosis entry. **a** Representative image of cells fixed without cold treatment (no cold) and those fixed after 10 min cold treatment are shown. For 10-min cold-treated cells, images are representative of those with kinetochore-microtubule (k-MT) and those with spindle microtubule remnants at the spindle pole (pole-MT, white arrow). The spindle was revealed by immunostaining for γ-tubulin (green), α-tubulin (red), and chromosomes (blue). **b** Mdivi-1-sensitized cells to cold-induced disassembly of spindle pole microtubules. The cells were treated with mdivi-1 as indicated, followed by 10 min cold treatment. Cells were then subjected to analysis of mitotic spindles. The percentages of cells containing the indicated types of microtubule are shown. Data are presented as mean ± SD from three independent experiments. *p < 0.05 comparing to 0 μM mdivi-1 by Student’s *t* test. **c** Images of Ro-3306-blocked-and-released mitotic cells. Representative images are shown of mitotic cells that were fixed immediately after release (0 min) with no spindle assembly and those fixed after release for 30 min with assembled spindles (30 min, Normal) or abnormal spindles (30 min, Abnormal). **d** Mdivi-1 disrupted bipolar spindle assembly rapidly after cells entered mitosis. Cells were first blocked with Ro-3306 and then released from the block for 30 min in the absence or presence of mdivi-1. The percentages of mitotic cells with the indicated types of the spindle are shown. Data are presented as mean ± SD from three independent experiments. *p < 0.05 comparing to 0 μM mdivi-1 by Student’s *t* test.

Since mdivi-1 may directly target tubulin and disrupt its polymerization, we further investigated how mdivi-1 affects the dynamic instability of microtubules, which is essential for mitotic spindle assembly^46^. To this end, we subjected untreated and mdivi-1-treated cells to cold treatment, which disassembles the cold-sensitive spindle pole microtubule (pole-MT) and preserves the cold-resistant kinetochore-microtubule (k-MT). As shown in Fig. 6a and b, 10-min cold treatment of mitotic cells without preceding mdivi-1 exposure disassembled the pole-MT and preserved the k-MT in about 75% of mitotic cells, while nearly 25% of the cells retained the pole-MT; importantly, exposures to 20 and 40 μM mdivi-1 before cold treatment, respectively, reduced the mitotic cells with retained pole-MT to 20% and 3%. These findings suggested that mdivi-1 may destabilize the pole-MT, and thus it dose-dependently sensitizes the cells to cold-induced disassembly of the pole-MT.

Given the essential roles of microtubule dynamic instability in mitotic spindle assembly and the effects of mdivi-1 we observed on microtubules, we decided to further test the acute effects of mdivi-1 on a spindle assembly. MDA-MB-231 cells were blocked by the CDK1 inhibitor Ro-3306 and released from Ro-3306 as described; the cells were then allowed to enter mitosis and assemble the spindle in the absence or presence of mdivi-1 for 30 min. As shown in Fig. 6c, 0 min, no assembled spindle microtubules were observed in mitotic cells fixed immediately upon Ro-3306 release. At 30 min after Ro-3306 release, up to 70% of the mitotic cells had assembled normal bipolar spindles in the absence of mdivi-1, leaving around 25% of cells that failed to assemble a normal bipolar spindle and exhibited abnormal spindles (Fig. 6c and d); thus, the majority of cells released from Ro-3306 readily assemble a bipolar spindle in the absence of mdivi-1. Importantly, when spindle assembly occurred in the presence of 20 or 50 μM mdivi-1, we respectively, observed 50% and 99% of the mitotic cells eventually exhibited abnormal spindles (Fig. 6c and d), indicating that mdivi-1 disrupts bipolar spindle assembly immediately after Ro-3306 release when cells enter mitosis. Taken together, our data indicated that mdivi-1 disrupts tubulin polymerization, induces microtubule destabilization, and may thus disturb the assembly of the mitotic spindle. These effects of mdivi-1, which are distinct from those of taxol, may further enhance the antimitotic effects of taxol and overcome taxol resistance.

## Discussion

TNBC is the most refractory subtype of breast cancer, and it is associated with chemoresistance, disease recurrence, and metastasis^1,3,47–49^. Although the tubulin-targeting taxanes, such as taxol, have been widely applied as first-line chemotherapeutics for breast cancers, drug side effects and tumor chemoresistance have limited their clinical application^2^. Thus, various combinations of taxol with other drugs are being tested in a broad effort to improve the efficacy of taxol-based chemotherapy^1,2^. In this study, we found that mdivi-1 enhances the antimitotic effects of taxol and can induce cytotoxicity in a taxol-resistant TNBC cell line, MDA-MB-231-TR. Our data thus imply that combining mdivi-1 with taxol could be a potentially effective strategy to improve the cytotoxic effects of taxol-based chemotherapy toward normal and resistant cancer cells.

We found that treatment of cells with mdivi-1 alone caused antimitotic effects, as it disrupted mitotic spindle assembly and induced mitotic arrest at 20–40 μM, in line with previously reported observations^36^. In that study, Wang et al. deduced that mdivi-1 may inhibit the integration of acentrosomal microtubule-organizing centers into genuine centrosomal spindle poles, thereby producing abnormal multipolar spindles. By measuring the intrinsic tryptophan fluorescence of tubulin and in vitro tubulin polymerization, the methods extensively applied to study tubulin-targeting drugs^50^, we demonstrated that mdivi-1 associates with tubulin and inhibits tubulin polymerization at 50–100 μM. The approximately two-fold higher effective mdivi-1 concentration in the in vitro assays than in the cells might be due to the potentially higher tubulin concentration used in the in vitro reactions which necessitates higher effective mdivi-1 concentration based on the stoichiometry of tubulin and mdivi-1. Secondly, the level of mdivi-1 might be enriched in the cells due to bioaccumulation, thus lowering the effective concentration used in the culture medium. Additional factors such as the different composition of tubulin isoforms and/or different post-translational modifications of tubulin exist in different model systems^51^, all of which could alter the effective range of drug concentrations. In spite of these issues, our results suggested that mdivi-1 at μM concentrations may directly bind to tubulin and inhibit tubulin polymerization both in vitro and in MDA-MB-231 cells in a dose-dependent manner. Moreover, similar dose ranges of mdivi-1 applied in the cells were found to sensitize spindle microtubules to cold-induced disassembly and to disrupt spindle assembly immediately after Ro-3306-blocked-and-released cells enter mitosis. These results indicated that mdivi-1 destabilizes the microtubule and acutely perturbs spindle assembly. Our data regarding microtubule destabilization by mdivi-1 thus may explain the previously reported formation of multiple acentrosomal spindle poles upon mdivi-1 treatment^36^; such destabilization would disrupt the dynamic instability of microtubules that is thought to be required for clustering of extra spindle poles into a bipolar spindle^52^. Furthermore, our data revealed a possible mechanism underlying the antimitotic effects of mdivi-1, wherein mdivi-1 directly associates with tubulin, inhibits tubulin polymerization, destabilizes microtubules and consequently perturbs mitotic spindle assembly. This mechanism would provide a straightforward explanation for the anti-proliferative activity of mdivi-1 in both MDA-MB-231 and MDA-MB-231-TR cells, as the mitotic errors would result in chromosome damage, cell cycle arrest, senescence, and apoptosis^53^.

The resistance to taxol can arise from overexpression of the ABC family of the drug efflux protein, which leads to the development of acquired multidrug resistance (MDR)^54^. Since our results showed that mdivi-1 displayed higher cytotoxicity in the taxol-resistant TR cells than in parental MDA-MB-231 cells, we reasoned that mdivi-1 might be a poor substrate for membrane MDR transporters and thus could be retained in TR cells to induce antimitotic effects. Alternatively, mutations at the taxol-binding site of tubulin or at microtubule-related proteins that can counteract taxol binding or balance taxol effects on microtubule stabilization and dynamics would also lead to cell resistance to taxol^55^. Based on the finding that mdivi-1 inhibited in vitro tubulin polymerization while taxol stabilized microtubules, we hypothesized that mdivi-1 might either act on tubulin via a mechanism distinct from taxol or bind to tubulin at sites different from those bound by taxol to exert its antimitotic effects. Thus, possible mutations or modifications occurring in TR cells that can counteract taxol effects fail to influence on the action mode of mdivi-1, resulting in the ability of mdivi-1 to overcome taxol resistance in TR cells. Further investigations are required to elucidate the precise mechanism(s) underlying TR resistance to taxol.

The hypothesis that mdivi-1 might bind to tubulin on different binding sites or act through different mechanisms from taxol may also explain the findings that mdivi-1 treatment further enhanced taxol-induced spindle abnormalities and cytotoxicity in MDA-MB-231 cells. Although the actual mdivi-1-binding sites on tubulin remain to be identified, the combined effects of mdivi-1 and taxol on spindle integrity and cytotoxicity let us to suspect that mdivi-1 may also potentiate taxol binding to tubulin in addition to its ability to inhibit tubulin polymerization, thus cooperatively altering microtubules functions. This hypothesis takes into consideration the previous findings that a catastrophe-inducing microtubule-targeting agent can increase the frequency of taxane binding to microtubules^56^. Notably, taxol and the microtubule-destabilizing drug, vinorelbine, have been found to cooperatively inhibit cancer cell proliferation^57^. These previous studies established the idea that microtubule destabilizing/depolymerizing agents may act to potentiate taxol binding to tubulin, enhancing microtubule perturbation and cytotoxicity. Further identification of an mdivi-1 binding site on tubulin and biochemical characterization of the mdivi-1–tubulin complex would improve the mechanistic understanding of how mdivi-1 inhibits tubulin polymerization and the interactions between mdivi-1, taxol, and tubulin.

Mdivi-1 was first identified from a chemical screen as an inhibitor of yeast Drp1, which induces mitochondrial elongation in both yeast and mammalian cells^29^. It has since been extensively used as a putative inhibitor of mammalian Drp1 and mitochondrial fission to address questions regarding mitochondrial function^58^. However, the inhibitory effect of mdivi-1 on Drp1 has been called into question by a later study^59^, which showed that mdivi-1 is a poor inhibitor of recombinant human Drp1 GTPase activity with a *K*_i_ > 1.2 mM. In addition, a number of studies have reported Drp1-independent cellular effects of mdivi-1^34–36,60^. These reports have motivated a reexamination of the literature^58^ and suggest that identification of other potential targets for mdivi-1 and elucidation of potential allosteric mechanisms of Drp1 inhibition are warranted. In our study, alterations in mitochondrial morphology and function were not obvious in mitotic cells containing mdivi-1-induced spindle abnormalities. Additionally, overexpression and depletion of Drp1 both failed to affect the extent of mdivi-1-induced spindle abnormalities and mitotic arrest. These findings led us to conclude that the antimitotic effects of mdivi-1 are probably independent of Drp1 and mitochondria. As an alternative mechanism, we found that mdivi-1 exhibits affinity to tubulin, implicating tubulin as a potential cellular target of mdivi-1. Since further biochemical characterizations of mdivi-1 are awaited, our data suggest future studies utilizing the molecule should include careful consideration and evaluation of how alterations in tubulin or microtubules may contribute to cellular outcomes.

In conclusion, our data demonstrate that mdivi-1 associates with tubulin and disrupts mitotic spindle assembly, independent of Drp1 and mitochondrial morphology and function. These actions may further enhance the antimitotic effects of taxol and overcome the taxol resistance in MDA-MB-231 cells. Thus, our data suggest the potential of mdivi-1 to improve the efficacy of taxol-based chemotherapy. Notably, various antitumor, neuroprotective, and cardioprotective effects have been demonstrated for mdivi-1^30,34–36,60–62^, and its therapeutic value continues to be explored. Given the side effects of tubulin-targeting drugs, such as peripheral neuropathy^2,63^, our data warrant further evaluation of microtubule-associated mechanisms in the development of mdivi-1-based therapeutic strategies.

## Materials and methods

### Cell culture and drug treatments

MDA-MB-231 cells were purchased from American Type Culture Collection and cultured as previously described^64^. To establish a taxol-resistant cell line, MDA-MB-231 cells were exposed to stepwise escalating taxol concentrations (from 0.5 to 4 nM), and resistant cells (MDA-MB-231-TR) were isolated and maintained in medium containing 4 nM taxol. The small molecule drugs used in this study include: mdivi-1 (M0199, Sigma Aldrich, St. Louis, MO, USA); tubulin-binding drugs, including taxol (paclitaxel, 580555, Merck, Darmstadt, Germany) and vinblastine (V1377, Sigma); and the CDK1 inhibitor, Ro-3306 (No. 15149, Cayman Chemical, Ann Arbor, MI, USA). To enrich mitotic cells, the cell cycle of MDA-MB-231 cells was blocked by 14-h treatment of 5 μM Ro-3306, followed by a PBS wash and 30-min incubation in drug-free medium to release the block and allow mitotic entry^65^. The mitotic cells then were shaken off culture plates and collected. Each drug was tested for their cytotoxicity in cells or for their effects in the in vitro system, followed by empirical adjustment to determine the appropriate treatment concentration. Cytotoxicity was measured with the trypan blue exclusion assay or colony formation assay, or by flow cytometry detection of the apoptosis marker, c-PARP, with a specific antibody (#9541, Cell Signaling, Danvers, MA, USA)^66^. Cell cycle analysis and detection of the mitosis marker, phospho-histone-H3, with a specific antibody (#9701, Cell signaling) were performed as described^66,67^.

### Depletion and overexpression of Drp1

The shRNAs targeting Drp1 (gene symbol *DNM1L*, TRCN-318424 and TRCN-318425) were purchased from the National RNAi Core Facility (Genomic Research Center, Academia Sinica). Depletion of endogenous Drp1 was performed using shRNAs prepared as described previously^64^. The expression vector for wild-type (WT) Drp1 was a gift from Gia Voeltz (Addgene plasmid # 49152)^68^ and the dominant negative pBABE-puro-hDrp1-Lys^38^ to Ala (K38A) was a gift from Christopher Counter and David Kashatus (Addgene plasmid # 37243)^42^. Cells stably expressing FLAG-tagged Drp1-WT or -K38A were established as described^64^. Cells expressing empty vector pLKO.1 and pFB-Neo were used as controls for depletion and overexpression of Drp1, respectively. The efficiency of depletion and overexpression was verified by Western blotting.

### Immunofluorescence staining and confocal microscopy

Cells seeded on coverslips were fixed, and immunofluorescence staining was performed as previously described^69^. The primary antibodies included anti-α-tubulin (GTX112141, GeneTex, Hsinchu, Taiwan or T5168, Sigma), anti-γ-tubulin (T6557 or T3559, Sigma), anti-pericentrin (ab4448 or ab28144, Abcam, Cambridge, UK) and anti-TOMM20 (ab56783, Abcam). Alexa-Fluor 488-, 568-, 633-, and 647-conjugated goat anti-mouse, as well as anti-rabbit IgG were purchased from Invitrogen (Carlsbad, CA, USA). Confocal images of the immunostained samples were obtained with a Leica TCS-SP5 microscope equipped with a HCX PLAPO ×63/1.4 objective at 300 Hz scanning speed; image stacks of 15–20 μm were collected with a 0.5-μm step size. The confocal image stacks were processed and maxima-projected in ImageJ for presentation.

### Analysis of mitotic spindles abnormalities

The morphology of mitotic spindles was revealed by immunostaining for α-tubulin and pericentrin as described above. The numbers of cells with indicated spindle types were counted using a Zeiss Axioplan 2 Imaging MOT fluorescence microscope. Mitotic spindles at prophases, prometaphases, and metaphases, judged as described previously^64,70^, were counted as normal spindles. Multipolar spindle, monopolar spindle, disorganized spindle, and bipolar spindle with misaligned chromosomes were judged as mitotic spindle abnormalities.

### Analysis of mitochondrial morphology and membrane potential

To analyze mitochondrial morphology, cells on coverslips were fixed and immunostained. Primary antibody against TOMM20, a mitochondria marker protein^41^, was followed by Alexa-Fluor 488-conjugated goat anti-mouse IgG, and then cells were imaged under a Leica confocal microscope (TCS-SP5). The change in mitochondrial morphology was determined by analyzing mitochondrial aspect ratios with Imaris. Briefly, the mitochondrial aspect ratio was calculated by creating a “surface” covering the continuous fluorescence signals of mitochondria marked by TOMM20. Then, the lengths of the longest and shortest axes of the ellipsoid (Fig. 4a right panel, yellow lines and ovals) circumscribing the mitochondria were measured, and the aspect ratio was calculated by dividing the longest axis length by the shortest axis length. A smaller aspect ratio suggests the mitochondrion is tuned toward fission. The distributions of mitochondrial aspect ratios during interphase and mitosis or in drug-treated cells were examined in at least 20 cells per group from two independent experiments.

To measure the mitochondrial membrane potential, MitoTracker Deep Red FM (M22426, Invitrogen), a mitochondrial membrane potential-dependent dye^44^, was utilized, following the protocol provided by the manufacturer. Cells stained with MitoTracker were counterstained with α-tubulin and then imaged under a Leica confocal microscope (TCS-SP5). Images were obtained under the same settings. The analysis of MitoTracker intensity was performed on Imaris. Briefly, a “spot” was created to cover a single cell and the total mitotracker intensity within the spot was measured. All the experiments were at least repeated three times.

### Analysis of mdivi-1 association with tubulin and in vitro tubulin polymerization assay

The interaction of mdivi-1 with tubulin was monitored in terms of intrinsic tryptophan fluorescence of tubulin^50^. When excited at 295 nm, tubulin displays a typical tryptophan emission spectrum with a maximum at 355 nm. Association of the drug to tubulin reduces the fluorescence emission. The affinity of mdivi-1 to tubulin was thus measured by detecting the emission spectrum of the intrinsic tryptophan fluorescence of tubulin, according to procedures described previously^45^. The reductions of fluorescence intensity at 355 nm at varying mdivi-1 concentrations were calculated, and the values were plotted on a double reciprocal plot. All the experiments were repeated at least three times.

A tubulin polymerization assay kit BK004P (Cytoskeleton Inc., Denver, CO, USA) was used according to the manufacturer’s instructions to examine the effects of mdivi-1 on in vitro tubulin polymerization. Briefly, 1 μl aliquots of mdivi-1 solubilized in DMSO at varying concentrations were added into 99 μl of the reaction mixture (3 mg/ml tubulin in 80 mM PIPES pH 6.9, 2 mM MgCl_2_, 0.5 mM EGTA, 1 mM GTP, 10.2% glycerol) in the wells of a 96-well plate. Then, the plate was immediately placed onto a pre-warmed chamber of a spectrophotometer (EnSpire™ Multilabel Plate Reader, PerkinElmer, Waltham, MA, USA), and the absorption at 340 nm was measured every 60 s for 1 h at 37 °C.

### Analysis of cellular tubulin polymerization and microtubule instability

To probe the effects of mdivi-1 on cellular tubulin polymerization, MDA-MB-231 cells were treated with mdivi-1 as indicated. Cells were then lysed and centrifuged as previously described^45^, and the polymerized tubulin fraction (in the pellet) and the soluble free tubulin fraction (in the supernatant) were subjected to western blotting analysis. Lysate from Ro-3306-blocked-and-released mitotic MDA-MB-231 cells, obtained as described above, was used as a control. The effect of mdivi-1 on the instability of microtubules was examined by cold treatment assay^71^. MDA-MB-231 cells were treated with mdivi-1 as indicated and subjected to 10 min cold treatment, followed by immunostaining, as described previously^64,70^. Mitotic cells with microtubule remnants at the spindle pole (pole-MT) were counted; a decrease in number indicates microtubule instability.

Ro-3306, a Cdk1-specific inhibitor, blocks cells at the G2-M transition, and after release from the block, cells can rapidly enter mitosis and assemble spindles^65^. The acute effect of mdivi-1 on spindle assembly thus was examined in MDA-MB-231 cells released from Ro-3306 block. Upon removal of Ro-3306, cells were left untreated or treated with mdivi-1 for another 30 min and then fixed and immunostained for mitotic spindles, as described above. The status of spindle assembly was then examined.

### Western blotting

Cell lysis and immunoblotting were performed as described^64^. Specific proteins were detected using antibodies against Drp1 (#8570, Cell Signaling, Denver, MA, USA), FLAG (F3165, Sigma), and α-tubulin. GAPDH was detected with anti-GAPDH (GTX100118, GeneTex) as a loading control.
